# Elevated fecal levels of the inflammatory biomarker calprotectin in early systemic sclerosis

**DOI:** 10.1007/s00296-022-05264-4

**Published:** 2022-12-25

**Authors:** Viggo Hamberg, Johan K. Wallman, Elisabeth Mogard, Elisabet Lindqvist, Tor Olofsson, Kristofer Andréasson

**Affiliations:** Department of Clinical Sciences Lund, Section of Rheumatology, Lund University, Skane University Hospital, 221 85 Lund, Sweden

**Keywords:** Systemic sclerosis, Gastrointestinal, Inflammation, Biomarker

## Abstract

Knowledge on gastrointestinal manifestations in early systemic sclerosis (SSc) is limited. We have investigated gastrointestinal inflammation in SSc at the time of diagnosis using the inflammatory biomarker Fecal calprotectin (F-cal). Consecutive patients with suspected SSc were characterized in relation to the 2013 classification criteria for SSc and classified as SSc or SSc-like disease. F-cal levels were measured with a polyclonal ELISA (Calpro A/S, Lysaker, Norway) and levels above 50 µg/g were considered elevated. F-cal levels were compared to those of control subjects without rheumatic disease. Of 137 patients with suspected SSc, 92 were classified as SSc and 45 as SSc-like disease. Median (interquartile range) disease duration among the SSc participants was 2.5 (1.2, 4.6) years. A substantial proportion of participants classified as SSc (35/92, 38%) and SSc-like disease (14/45, 31%) exhibited elevated F-cal compared to the control group (3/41, 7.3%; *p* < 0.001 and *p* = 0.007, respectively). Elevated F-cal was associated with proton pump inhibitor usage (OR 7.14; 95% CI 2.56–29.93; *p* < 0.001). We conclude that elevated F-cal is present in a subgroup of patients with SSc at the time of diagnosis, suggesting that that GI inflammation may be present in this patient group early in the disease course. F-cal did not exhibit potential to differentiate SSc from SSc-like disease.

## Introduction

Systemic sclerosis (SSc) is a systemic autoimmune disease characterized by a complex interaction of vasculopathy, immune dysregulation, and fibrosis [1]. Gastrointestinal (GI) manifestations are common, with prevalence estimates ranging up to 90%. GI manifestations have been attributed to a combination of smooth muscle atrophy and neural dysfunction, but the underlying pathology is still not well understood [2]. Yet, histopathological studies show that mural fibrosis and mucosal inflammation of the GI tract is present in this patient group [3].

GI manifestations have a profound and negative impact on health-related quality of life in the SSc population, and may fluctuate over time [4]. In addition to somatic discomfort, such as fecal incontinence, nausea, malnutrition and pain, GI morbidity may also indirectly affect quality of life negatively, causing social limitation or even depression [2, 5]. GI morbidity may develop during early stages of the disease course, causing irreversible tissue damage [6]. GI manifestations during early SSc have also been associated with increased mortality, partly because of malnutrition but also as a consequence of severe complications such as pseudo-obstruction [7].

Diagnosis of early SSc can be challenging. Both the 2013 American College of Rheumatology-European League Against Rheumatism (ACR-EULAR) classification criteria and the Very Early Diagnosis of Systemic Sclerosis (VEDOSS) criteria were partly defined to facilitate early diagnosis of SSc [8, 9]. GI items are not included in either set of criteria.

Calprotectin is a dimeric protein found in the cytoplasm of immune cells, especially neutrophils. It may be actively secreted or passively released during cellular turnover, and serves as a regulator of the innate immune response through the Toll-like receptor signaling pathway [10]. Calprotectin is a stable protein that can be measured in feces (F-cal). F-cal is an established sensitive and non-invasive marker of ongoing mucosal GI inflammation available for clinical use [10, 11]. F-cal has been reported to be elevated in patients with SSc when compared to both healthy controls and patients with other rheumatic diseases such as Sjögren’s syndrome and rheumatoid arthritis, and has also been associated with GI morbidity [12, 13]. However, F-cal has not been explored in early SSc and its diagnostic potential in the classification of SSc has not been studied.

The primary objective of this study was to investigate F-cal levels in recently diagnosed SSc. A secondary aim was to investigate the diagnostic potential of F-cal in the classification of SSc. An exploratory aim was to study F-cal in relation to GI features of early SSc.

## Methods

In this cross-sectional, observational study, all patients referred to the Department of Rheumatology at Skåne University Hospital, for in-house evaluation of newly onset SSc between January 1st, 2015, and December 31st, 2020, were considered for inclusion. All patients were referred to the SSc center by a senior rheumatologist because of suspected SSc. Subjects were invited to participate in the current study if they were able to produce a stool sample within ≤ 12 months of their initial evaluation at the clinic. Subjects were excluded from the study if they had previously been diagnosed with a non-SSc condition that is associated with elevation of F-cal. Conditions included in this definition were inflammatory bowel disease (IBD), GI malignancy, concurrent GI infection, recent GI surgery (≤ 4 weeks), or usage of permanent ostomy pouch.

Clinical characteristics were assessed at the first patient visit and collected retrospectively for all patients from the electronic hospital record charts. In addition, the following data were collected: age, sex, height, weight, disease subtype, serology, tobacco smoking, and disease duration defined as years since onset of first non-Raynaud’s Phenomenon (RP) disease manifestation. Gastrointestinal morbidity defined as the presence of reflux symptoms, dysphagia fecal incontinence and involuntary weight loss was collected by the physician through an in-house standardized protocol. Recent (within 1 week) use of proton pump inhibitors (PPI) or non-steroidal anti-inflammatory drugs (NSAIDs) and history of GI symptoms were noted. Esophageal dilation observed during high-resolution computed tomography (HRCT) was evaluated. Esophageal function was further investigated by barium cineradiography and classified on a scale from 0 (no involvement) to 3 (severe dysfunction/aperistalsis). All participants were examined in reference to the 2013 ACR-EULAR classification criteria, and those who fulfilled criteria were classified as “SSc patients”. Participants investigated for suspected SSc, but who did not fulfill the 2013 ACR-EULAR criteria, were classified as having “SSc-like disease”. Consequently, the group with SSc-like disease consisted of patients with new-onset symptoms indicative of SSc according to a senior rheumatologist, but who did not meet the 2013 classification criteria. These patients were further classified in reference to the VEDOSS criteria. Age- and sex-matched controls without rheumatic disease or IBD were recruited among hospital staff and their relatives or friends.

Stool samples were analyzed within 4 days of production, using a polyclonal Enzyme-Linked Immunosorbent Assay (ELISA) for detection of calprotectin (Calpro A/S, Lysaker, Norway). Laboratory analysis was performed according to the manufacturer’s instructions [14]. Briefly, stool samples were added to an extraction buffer 1:50 and subject to physical agitation. The extract was further diluted 1:100 in a dilution buffer, and 100 µl was then added to ELISA plate coated with affinity-purified monoclonal mouse antibodies specific for calprotectin. Calprotectin levels were measured by means of alkaline phosphatase-labeled rabbit antibodies against calprotectin. The ELISA had a lower detection limit of 30 µg/g. Calprotectin was defined as elevated if above 50 µg/g according to the manufacturer’s instructions and previous reports [15, 16].

For group comparison of categorical and dichotomous data, the χ^2^ or Fisher’s exact test was used, as appropriate. Receiver operating characteristic (ROC) curve analysis was performed and area under curve (AUC) was calculated. Odds ratios with 95% confidence intervals were calculated to estimate effect size. For group comparison of continuous and ordinal data, the Mann–Whitney *U* test was used. A significance level of α = 0.05 was used. All analyses were made using IBM SPSS Statistics version 27.0.0.0 and R version 4.1.1.

Ethical approval was obtained from the Regional Ethics Review Board, Lund, Sweden (reference number 2011/596) and written consent was provided by all patients and controls according to the Declaration of Helsinki.

## Results

In total, 178 participants were included in this study. Of these, 137 were patients with suspect newly onset SSc and 41 were healthy controls. Of the 137 patients, 92 were classified as SSc and 45 were classified as SSc-like disease. In all three groups, participants were of similar age and a similar proportion was female. Patient characteristics of these groups are shown in Table 1.

**Table 1:** Clinical characteristics and measurements of the study participants by group

	Suspected systemic sclerosis		Control, *N* = 41^a^
	Fulfilling 2013 classification criteria, *N* = 92^a^	Not fulfulling 2013 classification criteria (SSc-like disease), *N* = 45^a^	
Age (years)	54.2 (37.6, 65.9)	58.7 (44.4, 65.1)	51.0 (45.0, 58.0)
Disease duration (years)^b^	1.5 (0.75, 4.1)	0.6 (1.7, 3.6)^c^	NA
Female	78 (85%)	36 (80%)	34 (83%)
dcSSc	14 (15%)	NA	NA
2013 classification criteria			
Proximal scleroderma	30 (33%)	0 (0%)	NA
Puffy fingers	11 (12%)	9 (20%)	NA
Sclerodactyly	70 (76%)	3 (6.7%)	NA
Pitting scars	32 (35%)	0 (0%)	NA
Digital tip ulcer	10 (11%)	0 (0%)	NA
Telangiectasias	41 (45%)	6 (13%)	NA
Abnormal nailfold capillaries	79 (86%)	14 (33%)	NA
PAH	2 (2.2%)	0 (0%)	NA
ILD	36 (40%)	5 (12%)	NA
RP	90 (98%)	30 (67%)	NA
SSc-related autoantibodies	53 (58%)	12 (27%)	NA
Anticentromere	35 (38%)	12 (27%)	NA
Anti-topoisomerase I	15 (16%)	0 (0%)	NA
Anti–RNA polymerase III^d^	3 (3.7%)	0 (0%)	NA
Dysphagia	31 (34%)	16 (36%)	NA
Reflux symptoms	56 (62%)	20 (49%)	NA
Fecal incontinence	12 (13%)	1 (2.3%)	NA
Patient-reported involuntary weight loss	19 (21%)	15 (33%)	NA
Dilated esophagus on HRCT^e^	16 (21%)	3 (7.7%)	NA
Esophagus cineradiography severity			
None	38 (43%)	20 (49%)	NA
Low/Suspected	27 (30%)	10 (24%)	NA
Medium	13 (15%)	9 (22%)	NA
High	11 (12%)	2 (4.9%)	NA
Antinuclear antibodies^f^	81	32	NA
Homogenous pattern	18	8	NA
Speckled pattern	21	7	NA
Centromeric pattern	35	12	NA
Nucleolar pattern	19	4	NA
CRP (mg/L)	1.9 (1.0, 3.2)	1.9 (0.7, 8.3)	NA
BMI	25.7 (23.6, 27.2)	25.3 (22.8, 27.5)	NA
mRSS	2 (2, 7)	0 (0, 0)	NA
VC (% of predicted)	91 (78, 100)	89 (77, 102)	NA
NSAID use	18 (20%)	8 (18%)	NA
PPI use	52 (57%)	19 (42%)	NA
Fecal calprotectin (µg/g)^g^	35 (15, 80)	15 (15, 63)	15 (15, 15)
Fecal calprotectin > 50 µg/g	35 (38%)	14 (31%)	3 (7.3%)

*dcSSc* diffuse cutaneous systemic sclerosis, *PAH* pulmonary arterial hypertension, *ILD* interstitial lung disease, *RP* Raynaud’s phenomenon, *HRCT* high-resolution computed tomography, *NSAID* non-steroidal anti-inflammatory drugs, *PPI* proton pump inhibitor, *NA* not applicable

^a^Median (interquartile range) or *n* (%)

^b^Years after first non-Raynaud’s phenomenon manifestation

^c^missing data *n* = 2

^d^missing data *n* = 15

^e^missing data *n* = 20

^f^as evaluated with immune fluorescence. Some patterns may be overlapping

^g^ < 30 µg/g approximated to 15 µg/g

Among participants with confirmed SSc, median disease duration was 1.5 years. Diffuse cutaneous (dcSSc) subtype was diagnosed in 14/92 (15%) of these. Of patients with SSc-like disease, 30/45 (67%) exhibited RP, 14/43 (33%) exhibited pathological capillaroscopy, and 3/45 (6.7%) exhibited sclerodactyly. The VEDOSS criteria were met by 19/45 (42%) of these patients. Stool collection was made within 1 week of the initial clinical examination in 92% (127/138) of the study subjects.

**Fig. 1:**
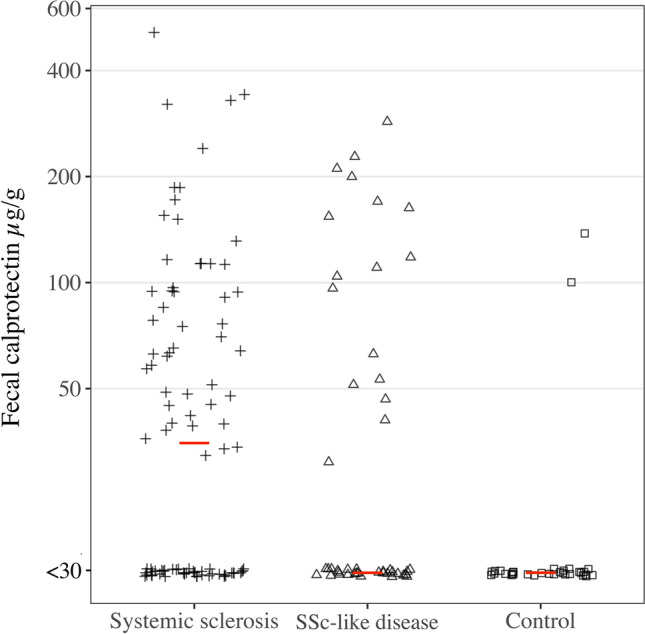
F-cal is elevated in SSc compared to controls, but not compared to patients with SSc-like disease. Individual F-cal measurements with median value marked by horizontal bar. Note logarithmic coordinates on *Y* axis. F-cal was significantly higher in the SSc group compared to the control group (*p* < 0.001) but not compared to patients with SSc-like disease (*p* = 0.248)

Of the SSc patients, 35/92 exhibited an elevated F-cal measurement. This was more common compared to the healthy control group, 3/41 (38 vs 7.3%; *p* < 0.001). Elevated F-cal was also more common in patients with SSc-like disease compared to the control group, 14/45 (31 vs 7.3%; *p* = 0.007; Fig. 1). The ROC analysis resulted in a maximum AUC of 0.728 (CI: 0.657–0.800) with a suggested optimal F-cal cut-off of 31.5 to differentiate SSc patients from healthy controls, yielding a specificity of 0.644 and a sensitivity of 0.533.

Similar proportions of patients with SSc and patients with SSc-like disease exhibited elevated F-cal (38 vs 31%; *p* = 0.427). Median F-cal did not significantly differ between these groups (35 vs < 30 µg/g; *p* = 0.248), indicating that F-cal was not able to discriminate between patients fulfilling the 2013 classification criteria and patients with SSc-like disease (Fig. 1). This was supported by ROC analysis, with an AUC of 0.557 (CI: 0.459–0.655) and a suggested optimal F-cal cut-off of 23.5 to differentiate SSc from SSc-like, yielding a specificity of 0.939 and sensitivity of 0.533. Elevated F-cal levels were seen in 2/19 (11%) of the subgroup fulfilling the VEDOSS criteria.

**Fig. 2:**
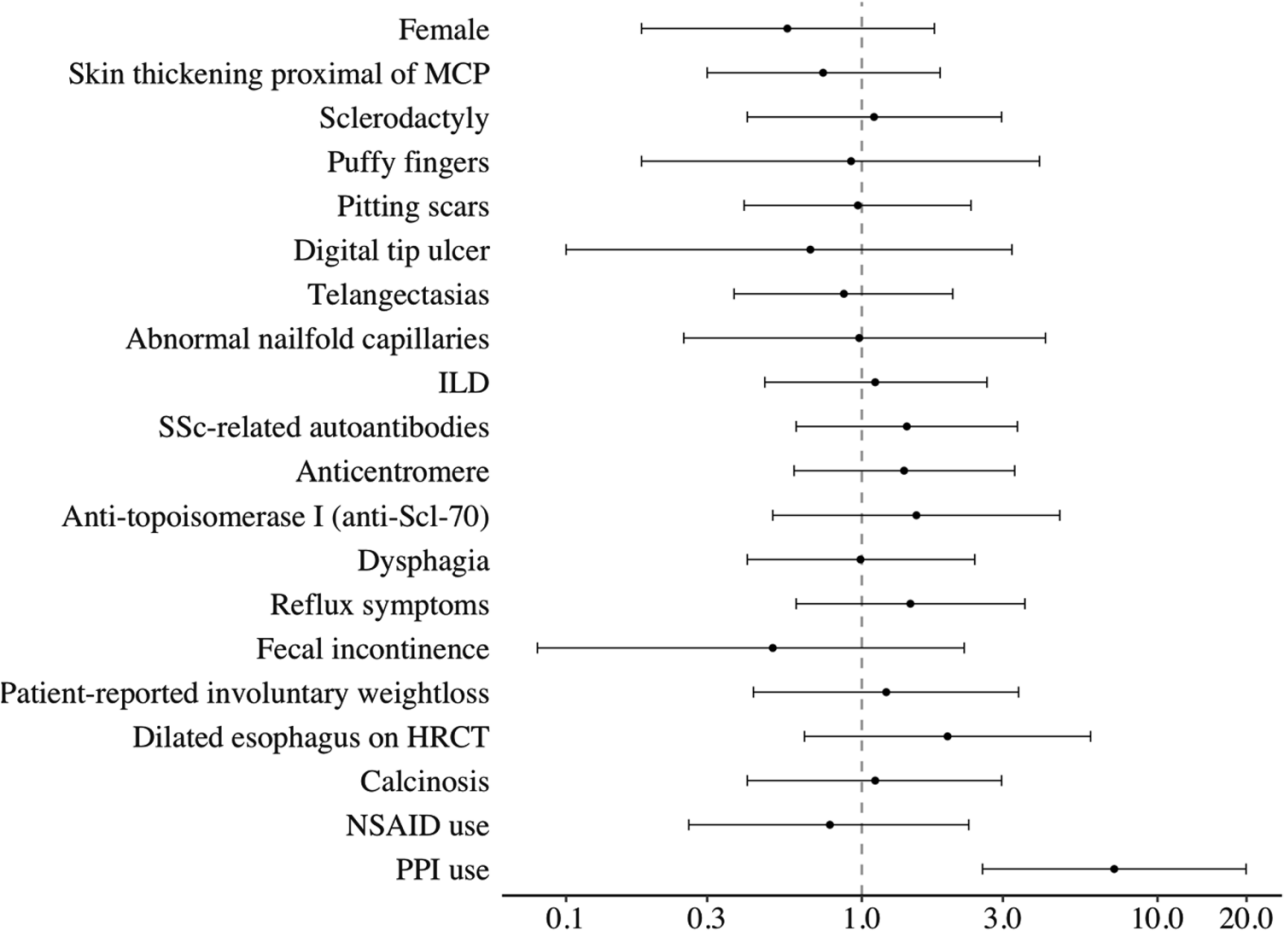
Plot of odds ratios of elevated F-cal in patients with newly diagnosed systemic sclerosis. Odds ratio of having elevated F-cal by patient characteristic in patients fulfilling the 2013 classification criteria for systemic sclerosis. Note logarithmic coordinates on *X* axis. Odds ratio marked by point and 95% confidence interval marked by error bars. Patients using proton pump inhibitors had increased odds of exhibiting elevated F-cal levels. *MCP* metacarpophalangeal joints, *ILD* interstitial lung disease, *HRCT* high-resolution computed tomography, *NSAID* non-steroidal anti-inflammatory drugs, *PPI* proton pump inhibitor

Among the SSc patients, elevated F-cal did not associate with clinical features of disease including esophageal dilatation, disease duration or the investigated GI symptoms (reflux, dysphagia, fecal incontinence, and involuntary weight loss). Also, we did not find any association between esophageal dysmotility and F-cal (*p* = 0.444). PPI use was significantly more common in SSc patients who had an elevated F-cal measurement compared to those who did not (OR 7.14; 95% CI 2.56–29.93; *p* < 0.001, Fig. 2).

## Discussion

In this cross-sectional study, we show that calprotectin levels in feces are elevated in a substantial proportion of SSc patients compared to healthy controls already at the time of diagnosis. These results are in agreement with the hypothesis that GI inflammation may be an early manifestation of SSc [6] Calprotectin has been established as a marker of inflammation and neutrophil activation in several diseases, including SSc [17]. Recently, calprotectin dependent neutrophil mediated inflammation has been described in relation to disease pathogenesis in SSc, further promoting the rationale of exploring calprotectin as biomarker in SSc [18]. Our study are in line with these reports, as 35 out of 92 patients exhibited elevated levels of F-cal.

In this study, we also show that F-cal lacks discriminatory power to separate SSc, as defined by the 2013 ACR-EULAR criteria, from SSc-like disorders in a diagnostic tertiary referral setting. One reason for this may be that patients with SSc-like disorders, including VEDOSS, experience GI inflammation at similar rate as patients classified as SSc. GI symptoms were not associated with elevated F-cal in this study. This is partly in agreement with previous studies suggesting that GI inflammation does not necessarily correlate with self-reported GI morbidity in SSc [19]. However, these results differ from studies assessing SSc GI symptoms using the University of California Gastrointestinal Rating Scale [13]. Unfortunately, this questionnaire, developed specifically for SSc, was not used in this study as we were not aware of a Swedish translation at the study inception.

In this study, F-cal elevation was not associated with radiological findings of esophageal disease, which differs from previous studies [12, 13]. It is possible that the short disease duration of patients investigated here may account for these results, especially if GI inflammation is thought to precede GI dysmotility over the disease course. It should also be noted that calprotectin in stool may originate from both the upper and the lower GI tract [20].

PPI use was significantly associated with elevated F-cal in SSc patients. This association has also been reported in other cohorts [21]. One possible explanation is that patients with GI inflammation are more likely to use PPIs. Another possible explanation is that PPI use may cause increased levels of F-cal, although such a mechanism remains unclear [22].

Early diagnosis of SSc remains a clinical challenge, and GI abnormalities have previously been reported in patients with suspect SSc not fulfilling the 2013 ACR-EULAR criteria [23]. In addition, GI pathologies have been presented as independent risk factors for VEDOSS patients to develop SSc [24]. It remains to be explored to what degree pathological F-cal testing can predict future development of SSc.

One limitation of our study is that patients with elevated F-cal levels were not investigated endoscopically, and we were consequently unable to evaluate if the F-cal originated from inflamed mucosa. Another limitation is the cross-sectional study design, wherein we do not know if patients with increased F-cal later developed GI symptoms, or vice versa. Selection bias may also pose an issue, as patients experiencing GI discomfort may be less inclined to provide voluntary stool samples. A strength of this study is the relatively large group of well-characterized participants with new-onset SSc and SSc-like disease.

In conclusion, we report that a substantial proportion of patients with SSc exhibit elevated F-cal levels already at the time of diagnosis, suggesting that GI inflammation is present in early stages of this disease. F-cal could not discriminate SSc from SSc-like diseases in a tertiary diagnostic setting. The pathophysiology and time course of GI manifestations of SSc need further exploration.

## Previous publication

Some of the results reported in this manuscript has previously been published as a congress abstract for the EULAR Congress 2022 [25].

## Data Availability

Unidentified patient data are available upon reasonable request at: viggo.hamberg@gmail.com.
